# Assessment of body composition and association with clinical outcomes in patients with lung and colorectal cancer

**DOI:** 10.1259/bjro.20210048

**Published:** 2021-11-26

**Authors:** Naomi S Sakai, Anisha Bhagwanani, Timothy JP Bray, Margaret A Hall-Craggs, Stuart Andrew Taylor

**Affiliations:** 1 UCL Centre for Medical Imaging, London, UK; 2 University College London Hospital, London, UK

## Abstract

**Objectives:**

To assess body composition in patients with non-small cell lung cancer (NSCLC) and colorectal cancer using whole-body MRI and relate this to clinical outcomes.

**Methods:**

53 patients with NSCLC (28 males, 25 females; mean age 66.9) and 74 patients with colorectal cancer (42 males, 32 females; mean age 62.9) underwent staging whole-body MRI scans, which were post-processed to derive fat mass (FM), fat free mass (FFM) and skeletal muscle (SM) indices and SM fat fraction (FF). These were compared between the two cancer cohorts using two-sided *t*-tests and the chi-squared test. Measurements of body composition were correlated with outcomes including length of hospital stay, metastatic status and mortality.

**Results:**

Patients with NSCLC had significantly lower FFM (*p* = 0.0071) and SM (*p* = 0.0084) indices. Mean SM FF was greater in patients with NSCLC (*p* = 0.0124) and was associated with longer hospital stay (*p = 0.035*). There was no significant relationship between FM, FFM and SM indices and length of hospital stay, metastatic status or mortality.

**Conclusions:**

Patients with NSCLC had lower FFM and SM indices than patients with colorectal cancer and greater SMFF, indicating lower SM mass with fatty infiltration. These findings reflect differences in the phenotype of the two groups and suggest patients with lung cancer are more likely to require additional nutritional support.

**Advances in knowledge:**

Body composition differs between NSCLC and colorectal cancer. Patients with NSCLC have both a reduced SM mass and greater SM FF suggesting that they are more nutritionally deplete than patients with colorectal cancer.

## Introduction

Patients with cancer are at high risk for malnutrition both as a result of the disease itself and the associated treatments. Malnutrition is defined as a state resulting from lack of intake or uptake of nutrition that leads to altered body composition, and it is known to result in poorer clinical outcomes from disease.^1^ In patients with cancer, malnutrition results from both reduced food intake and metabolic disturbances, which are provoked by the activation of systemic inflammation.^1^ The combination of loss of appetite and tissue breakdown leads to substantial loss of body weight, alterations in body composition and decreased functional capacity.

It is estimated that 10–20% of deaths in patients with cancer are a result of malnutrition rather than the malignancy itself.^2,3^ Change in body composition, specifically the loss of skeletal muscle (with or without the loss of fat), is thought to be more important than weight loss in isolation. Loss of muscle mass and function is termed sarcopenia, defined as muscle mass more than two standard deviations below the reference value.^4^ Sarcopenia in cancer patients is associated with a greater incidence of complications after surgery,^5,6^ increased treatment toxicity^7,8^ and decreased survival.^9,10^ Sarcopenia is particularly important to identify in obese patients (*i.e.,* those with sarcopenic obesity) because it is independently associated with higher mortality and rates of complications.^11^ Whilst one in four patients with obesity are sarcopenic, the diagnosis is often overlooked given the elevated BMI.^12^

The association between body composition and outcomes has been investigated in cancers including colorectal,^13^ breast,^14^ gastrointestinal^15^ and prostate.^16^ However, differences in the body composition of patients have not been compared across cancer types, and differences in outcomes related to body composition between cancer types are not well defined.

Assessment for malnutrition in cancer patients is important because of the adverse outcomes and because sarcopenia and sarcopenic obesity can be treated. There are guidelines to enable identification, prevention and treatment of malnutrition in cancer patients^17^ with the most effective interventions being combinations of physical exercise and adequate protein intake.^18^

Most of the research to date on the effect of body composition on outcomes in patients with cancer has used CT to measure body composition. MRI is increasingly used in cancer staging and has been shown to have comparable accuracy to standard pathway staging imaging (usually CT or PET-CT).^19,20^ In this study, body composition was measured using MRI in patients with colorectal and non-small cell lung cancer (NSCLC). The body composition of the two cohorts was compared and the association between body composition and clinical outcomes investigated.

## Methods

This study uses data from the Streamline C^19^ and Streamline L^20^ trials, which are registered with the International Standard Randomised Controlled Trial registry, numbers ISRCTN43958015 and ISRCTN50436483, respectively. The study was granted ethical approval on 3 October 2012. All patients gave written informed consent.

### Patients

Patients with colorectal or NSCLC were prospectively recruited from 16 sites as part of two studies examining the utility of whole-body MRI in cancer staging compared to standard staging pathways (Streamline C and Streamline L). Six imaging hubs performed the whole-body MRI. Inclusion criteria for the Streamline C trial were: age ≥18 with histologically proven or suspected colorectal cancer referred for staging. Suspicion of colorectal cancer was defined as the presence of a mass on endoscopy or imaging (or both), triggering staging investigations. Inclusion criteria for the Streamline L trial were: age ≥18 with suspected primary NSCLC on chest CT or histologically proven primary NSCLC, potentially radically treatable disease defined as stage IIIb or less on diagnostic CT, performance status 0–2.

370 and 353 patients were recruited to Streamline C and Streamline L, respectively. 299 (Streamline C) and 187 (Streamline L) patients completed the studies. Patients were included in the current study if they were scanned at the lead imaging hub which included quantitative chemical shift-encoded (CSE)-MRI sequences as part of their MRI scan protocol, and their height and weight were recorded. Only patients from the lead institution were included as other sites did not use CSE sequences. Patients were excluded if the MR images were of poor quality such that body composition analysis was deemed not possible.

All patients were followed up for 12 months or until death (if sooner).

Age and sex were collected from the Streamline study databases. Electronic healthcare records were examined for height and weight information. Body mass index (BMI) was calculated from the patients’ height and weight:

$$\begin{array}{c} BMI=\frac{weight\;\left(kg\right)}{\;{\left(height\;\left(m\right)\right)}^{2}}. \end{array}$$

Length of hospital stay (in days) for each patient over the 12 month follow up period was collected from the study databases. The number of days was summed if there was more than one episode to give the total length of hospital stay. For patients who underwent surgery, the total length of hospital stay was subcategorised to give the number of days directly related to surgery.

As part of the main study protocol, a multidisciplinary consensus panel review was used to assign metastatic status for each patient at the time of recruitment, using all available imaging, histological and clinical data over the 12-month follow-up period.^19,20^ Metastatic status at the time of recruitment was summarised as either ‘yes’ or ‘no’.

Performance status data were collected from the study databases. These were categorised according to the WHO performance status classification.^21^

### Imaging acquisition

Patients underwent a whole-body MRI scan (from the cranial vertex to mid-thigh) with conventional and quantitative CSE-MRI (Dixon). All MRI scans were performed on a 3T Philips Ingenia system (Ingenia, Philips, Amsterdam, Netherlands). CSE-MRI was performed using a vendor-supplied two-point gradient echo sequence (Philips mDixon). Images were acquired in the coronal plane with the following acquisition parameters: flip angle 10°, first echo time 1.02 ms, echo spacing 1 ms, repetition time 18 ms, matrix size 480 × 480, resolution 2 × 2 × 5 mm^3^.

Additional sequences acquired included axial diffusion-weighted imaging (single shot echo planar readout, b-values 50 and 900 s/mm^2^, slice thickness 5 mm), axial *T*_2_-weighted turbo spin echo (TR/TE 934-1121/80 ms, slice thickness 5 mm), and coronal post-contrast Dixon images.

### Post-processing

**Figure 1.**
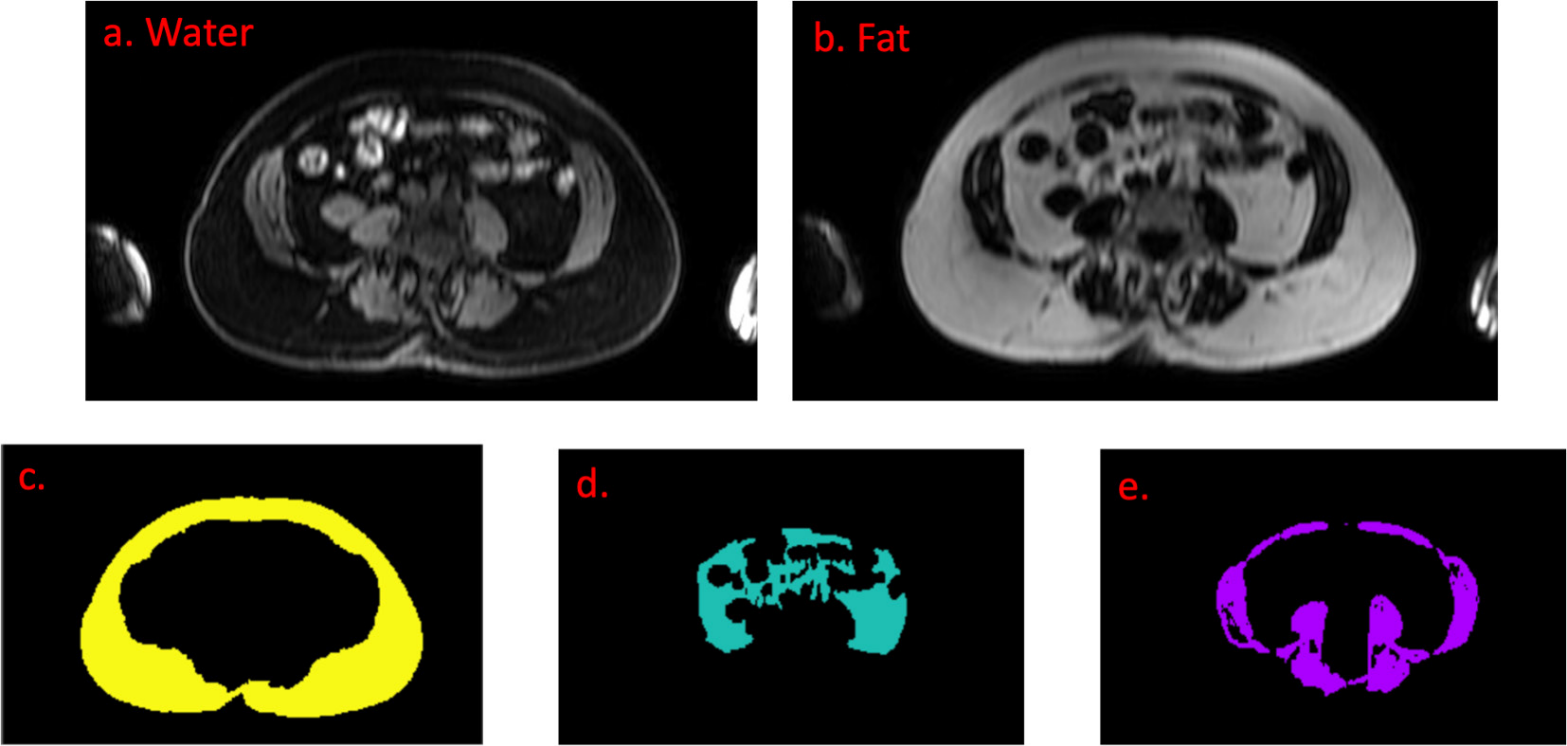
Input water (a) and fat (b) images are used to segment (c) subcutaneous fat, (d) visceral fat and (e) skeletal muscle

Images were post-processed using an in-house tool for segmenting adipose tissue and skeletal muscle (see Supplementary Material 1). The tool uses paired axial fat-only and water-only images at the level of the L3 vertebral body as input data. Examples of input MR images and segmented areas of subcutaneous fat, visceral fat and skeletal muscle are shown in Figure 1.

Regions of interest (ROIs) were generated by two radiology registrars (NS and AB, each with three years of experience in body MRI), blinded to the diagnosis, to segment the visceral and subcutaneous adipose tissue and the skeletal muscle (including abdominal wall, paraspinal and psoas muscles) at the level of the L3 vertebral body.

Total body fat mass (FM) and fat-free mass (FFM) were estimated according to regression equations previously published by Mourtzakis (2018). ^22^

$$\begin{array}{c} Total\;body\;FM\left(kg\right)=0.042\times \left[total\;adipose\;tissue\;at\;L3\left(cm^{2}\right)\right]+11.2; \end{array}$$

$$\begin{array}{c} Total\;body\;FFM\;\left(kg\right)=0.3\times \left[skeletal\;muscle\;at\;L3\left(cm^{2}\right)\right]+6.06. \end{array}$$

FM and FFM were normalised for stature to derive the FM index (kg/m^2^) and FFM index (kg/m^2^), respectively:

$$\begin{array}{c} FM\;index\;\left(kg/m^{2}\right)=\frac{Total\;body\;FM}{height\left(m^{2}\right)}; \end{array}$$

$$\begin{array}{c} FFM\;index\left(kg/m^{2}\right)=\frac{Total\;body\;FFM}{height\left(m^{2}\right)}. \end{array}$$

Sarcopenia was defined based on a previous study performed in patients with cancer: L3 skeletal muscle (SM) index (total L3 skeletal muscle mass normalized for stature)≤38.5 cm^2^/m^2^ for females and ≤52.4 cm^2^/m^2^ for males.^23^ Sarcopenic obesity was defined as the presence of both sarcopenia and obesity (BMI ≥30 kg/m^2^).

SM signal fat fraction (FF) was calculated as:

.$$signalFF(\%)=\frac{sFat}{sFat+sWater}$$

### Statistical analysis

Normality of data was confirmed using the D’Agostino-Pearson omnibus test. Baseline characteristics between patients with colorectal cancer and those with NSCLC were compared using two-sided *t*-tests and the chi-squared test. Differences in measures of body composition were compared using two-sided *t*-tests.

Linear regression was used to assess the relationship between body composition parameters measured using MRI and baseline characteristics (sex, age, BMI).

Linear regression was used to assess the relationship between the length of hospital stay and body composition parameters measured using MRI. The relationship between metastatic status and mortality and body composition was assessed using 2-sided *t*-tests.

## Results

96 patients with colorectal cancer were identified, with nine excluded due to missing height or weight information and 13 due to suboptimal MRI scan quality. 68 patients with NSCLC were identified, with three excluded due to missing height or weight information and 12 due to suboptimal MRI scan quality. In total, 127 patients were included in the study: 74 with colorectal (42 males and 32 females; mean age 62.9 ± 12.1 [mean age in years ± SD]) and 53 with NSCLC (28 males and 25 females; mean age 66.9 ± 10.5 [mean age in years ± SD]).

There were no significant differences in baseline characteristics between the two patient cohorts Table 1.

**Table 1.** Baseline characteristics of the patient cohorts

	Colorectal *n* = 74	NSCLC *n* = 53	
**Sex** (M : F)	42 : 32	28 : 25	χ^2^=0.1925 *p* = 0.6609
**Age in years** (mean ± SD)	62.9 ± 12.1	66.9 ± 10.5	*p* = 0.0592
**BMI in kg/m^2^** (mean ± SD)	26.0 ± 4.12	25.1 ± 4.59	*p* = 0.2865
**Metastatic disease at diagnosis**	19 (26%)	13 (25%)	χ^2^=0.02157 *p* = 0.8832

NSCLC, non-small cell lung cancer; SD, standard deviation.

### Body composition

#### Comparison between NSCLC and colorectal cancer patients

**Figure 2.**
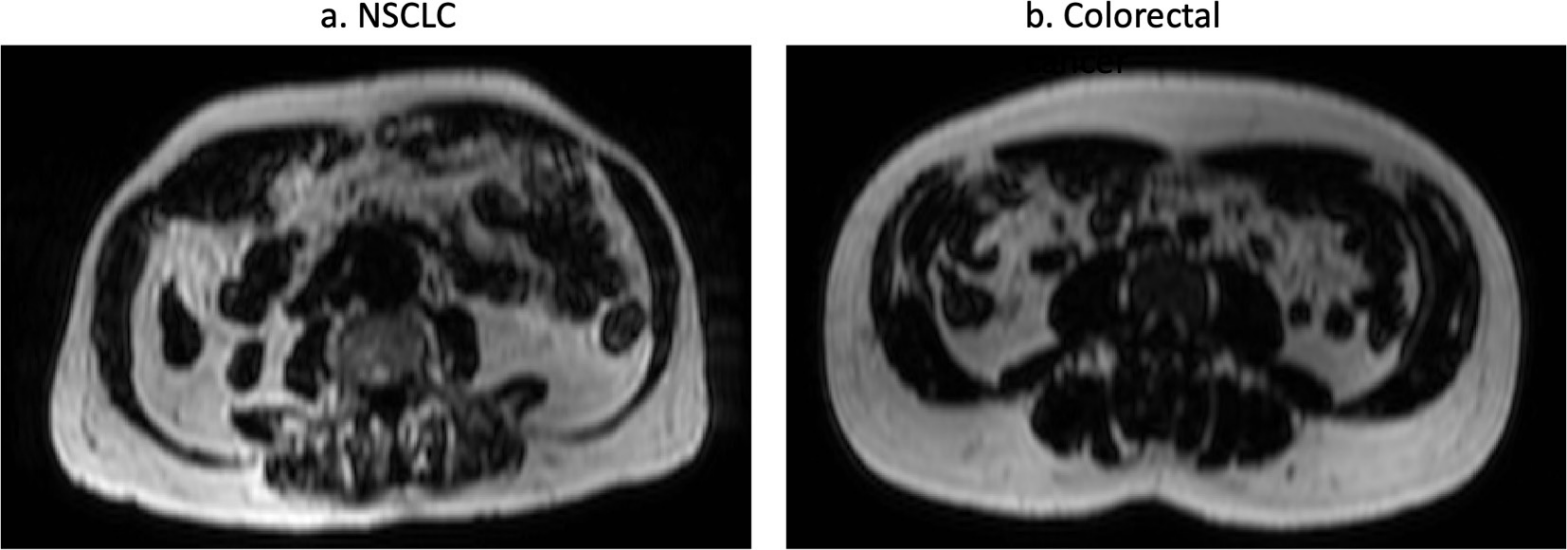
Example axial fat-only images at the level of the L3 vertebral body in patients with (a). NSCLC and (b). colorectal cancer. These patients have similar FM indices but different FFM and SM indices. (NSCLS FM index 8.3, FFM index 15.1, SM index 43.2, SM FF 45.3%; colorectal cancer FM index 7.9, FFM index 21.1, SM index 63.9, SM FF 28.6%). Abbreviations: NSCLC non-small cell lung cancer, FM fat mass, FFM fat free mass, SM skeletal muscle

Patients with NSCLC had significantly lower FFM (*p* = 0.0071) and SM (*p* = 0.0084) indices than those with colorectal cancer. Mean SM FF was greater in patients with NSCLC than those with colorectal cancer (*p* = 0.0124), Table 2. Example images from two patients are shown in Figure 2.

**Table 2.** Comparison of body composition parameters between colorectal and lung cancer groups. Results are displayed as mean ± SEM

	Colon mean *n* = 74	NSCLC mean *n* = 53	*Sig.*
**FM index** (kg/m^2^)	8.881 ± 0.2912	8.597 ± 0.3270	0.5202
**FFM index** (kg/m^2^)	17.64 ± 0.3269	16.28 ± 0.3716	0.0071 **
**SM index** (kg/m^2^)	51.52 ± 1.082	47.09 ± 1.237	0.0084 **
**Muscle FF** (%)	36.24 ± 0.9692	40.19 ± 1.247	0.0124 *
**Sarcopenic : not sarcopenic**	19 : 55	22 : 31	0.0598

FF, fat fraction; FFM, fat free mass; FM, fat mass; NSCLC, non-small cell lung cancer; SEM, standard error of the mean; SM, skeletal muscle.

Significance is summarised as follows: *p ≤ 0.05, **p ≤ 0.01, ***p ≤ 0.001.

19 (26%) patients with colorectal cancer were sarcopenic compared with 22 (42%) with NSCLC. 3 (6%) patients with NSCLC and no patients with colorectal cancer had sarcopenic obesity.

Performance status data were missing for 8/53 patients with NSCLC and 27/74 patients with colorectal cancer. Of those patients for whom performance status was available, patients with colorectal cancer had a lower mean performance status (which equates to greater preservation of physical activity) than those with NSCLC, 0.19 *vs* 0.45, *p* = 0.0121. Patients with colorectal cancer who underwent surgery had a lower mean performance status than those who did not undergo surgery (0.14 *vs* 0.60, *p* = 0.0134); there was no significant difference in the performance status of patients with NSCLC who underwent surgery compared to those who did not (0.60 *vs* 0.36, *p* = 0.176). There was no significant difference in the mean performance status of patients who were or were not sarcopenic for either colorectal cancer or NSCLC (colorectal 0.36 *vs* 0.14, *p* = 0.102; NSCLC 0.62 v. 0.33, *p* = 0.104).

#### BMI and MRI measurements of body composition

There was a significant positive relationship between BMI and all the body composition parameters. The relationship between BMI, FM index, FFM index, SM index and SM FF is summarised in Supplementary Table A1.

### Clinical outcomes

#### Length of hospital stay

65 patients with colorectal cancer (87.8%) and 27 patients with NSCLC (50.9%) underwent surgery. Data on length of hospital stay were unavailable for two patients with colorectal cancer and six patients with NSCLC.

Mean length of hospital stay related to surgery was greater in patients with colorectal than NSCLC (11.7 ± 1.313 days compared with 7.0 ± 0.7496 days, respectively, *p* = 0.0463). Mean total stay for all patients (including those who did not have surgery) was greater in patients with colorectal cancer than NSCLC (10.2 ± 1.236 days compared with 5.63 ± 1.089 days respectively, *p* = 0.0099), Supplementary Table A2.

Mean length of hospital stay for patients with and without sarcopenia for the two cancer cohorts is summarised in Table 3. In patients with colorectal cancer, there was no difference in the mean length of hospital stay in patients with and without sarcopenia. In patients with NSCLC, there was a significantly greater mean total length of hospital stay (including all patients) in patients who were not sarcopenic (7.71 ± 1.52 days) compared with those who were sarcopenic (2.91 ± 1.35 days), *p* = 0.027.

**Table 3.** Mean length of hospital stay for patients with and without sarcopenia for the two cancer cohorts. Length of hospital stay is shown as (a) that related to surgery and (b) the total for all patients. Averages are displayed as mean ± SEM

	Sarcopenic	Not sarcopenic	*Sig.*
**Colorectal**	*n* = 19	*n* = 55	
**Surgical length of hospital stay** (days)	11.06 ± 1.71	11.96 ± 1.72	0.760
	*n* = 18	*n* = 45	
**Total length of hospital stay** (days)	14.39 ± 2.27	15.24 ± 2.92	0.871
	*n* = 18	*n* = 54	
	**Sarcopenic**	**Not sarcopenic**	***Sig.***
**NSCLC**	*n* = 22	*n* = 31	
**Surgical length of hospital stay** (days)	6.17 ± 1.92	7.33 ± 1.68	0.496
	*n* = 6	*n* = 15	
**Total length of hospital stay** (days)	2.91 ± 1.35	7.71 ± 1.52	0.027 *
	*n* = 21	*n* = 28	

NSCLC, non-small cell lung cancer; SEM, standard error of the mean.

Significance is summarised as follows: * *p* ≤ 0.05, ** *p* ≤ 0.01, *** *p* ≤ 0.001.

The relationship between length of hospital stay and the body composition parameters for the two cohorts is summarised in Tables 4 and 5 (a summary of these results separately for each sex has been provided in the Supplementary Tables A3 and A4). In patients with colorectal cancer who underwent surgery, there was a positive relationship between FFM and SM indices and the length of hospital stay. This relationship was not seen when looking at the total length of hospital stay (for all reasons for hospital admission, for all patients). In patients with NSCLC, there was a positive relationship between both the length of hospital stay related to surgery and SM FF and the total length of hospital stay and SM FF *p* = 0.035.

**Table 4.** Summary of the regression analysis for length of hospital stay and body composition parameters for patients with colorectal cancer. Length of hospital stay is shown as (a) that related to surgery and (b) the total for all patients

Colorectal			
	*B*	*SE B*	*Sig.*
**Surgical Length of Hospital Stay**			
**FM index** (kg/m^2^)	−0.196	0.524	0.709
**FFM index** (kg/m^2^)	0.946	0.459	0.043 *
**SM index** (kg/m^2^)	0.299	0.138	0.034 *
**Muscle FF** (%)	−0.029	−0.165	0.886
**BMI** (kg/m^2^)	−0.134	0.316	0.673
**Total Length of Hospital Stay**			
**FM index** (kg/m^2^)	−0.291	0.914	0.751
**FFM index** (kg/m^2^)	−0.538	0.828	0.518
**SM index** (kg/m^2^)	−0.136	0.251	0.588
**Muscle FF** (%)	0.238	0.275	0.389
**BMI (**kg/m^2^)	−0.080	0.551	0.885

BMI, body mass index; FF, fat fraction; FFM, fat free mass; FM, fat mass; SM, skeletal muscle.

Significance is summarised as follows: *p ≤ 0.05, **p ≤ 0.01, ***p ≤ 0.001.

**Table 5.** Summary of the regression analysis for length of hospital stay and body composition parameters for patients with NSCLC. Length of hospital stay is shown as (a) that related to surgery and (b) the total for all patients

NSCLC			
	*B*	*SE B*	*Sig.*
**Surgical Length of Hospital Stay**			
**FM index** (kg/m^2^)	0.566	0.326	0.098
**FFM index (**kg/m^2^)	−0.084	0.263	0.752
**SM index (**kg/m^2^)	−0.033	0.077	0.677
**Muscle FF** (%)	0.176	0.060	0.009 **
**BMI (**kg/m^2^)	0.202	0.168	0.243
**Total Length of Hospital Stay**			
**FM index** (kg/m^2^)	0.801	0.450	0.082
**FFM index (**kg/m^2^)	0.443	0.416	0.293
**SM index (**kg/m^2^)	0.104	0.125	0.409
**Muscle FF** (%)	0.252	0.116	0.035 *
**BMI (**kg/m^2^)	0.336	0.236	0.162

BMI, body mass index; FF, fat fraction; FFM, fat free mass; FM, fat mass; NSCLC, non-small cell lung cancer; SM, skeletal muscle.

Significance is summarised as follows: *p ≤ 0.05, **p ≤ 0.01, ***p ≤ 0.001.

#### Metastatic status

There was no significant difference in the body composition parameters measured using MRI (FM index, FFM index, SM index and muscle FF) and metastatic status at recruitment for the two groups, Supplementary Table A5.

#### Mortality

Seven patients died in each group within 12 months. There was no significant difference in mortality between the colorectal cancer and NSCLC groups (*p* = 0.506).

There was no significant difference in the body composition parameters measured using MRI and mortality at 12 months for the two groups, Supplementary Table A6.

## Discussion

This study investigated the use of MRI to measure body composition in patients with colorectal cancer and NSCLC and the relation of body composition to clinical outcomes. BMI was positively associated with all of the measured body composition parameters (FM index, FFM index, SM index and SM FF). Patients with NSCLC had lower FFM and SM indices than patients with colorectal cancer, indicating that they have a lower SM mass. In addition, the SM FF was greater in patients with NSCLC suggesting fatty infiltration or replacement. These are new findings and suggest that patients with lung cancer are more nutritionally deplete than patients with colorectal cancer.

Patients with colorectal cancer had, on average, longer hospital stays than those with NSCLC. This includes both the total stay for all patient episodes and the length of hospital stay directly related to surgery (for those who underwent surgery). The greater length of total hospital stay amongst patients with colorectal cancer may therefore be attributable to the longer stays experienced by those having surgery. A much larger proportion of the colorectal cancer patients had surgery than those with NSCLC. There are several possible explanations for this finding: treatment for colorectal cancer is more likely to involve primary surgery, whereas NSCLCs may be treated with (chemo)radiotherapy or palliation depending on tumour stage and patient comorbidities (patients with NSCLC are more likely to have smoking-related comorbidities such as chronic obstructive pulmonary disease and cardiovascular disease that increase the risk of general anaesthesia and surgery).^24^

Low muscle mass and sarcopenia have previously been shown to be associated with poorer surgical outcomes (including increased length of hospital stay and post-operative complications) in numerous cancers.^25^ Many previous studies have used CT to measure body composition because this is acquired as part of routine staging. A Canadian study of 234 patients undergoing surgery for colorectal cancer measured total skeletal muscle at the level of the L3 vertebral body on pre-operative CT.^26^ In the 38.9% of patients who were sarcopenic, length of hospital stay was longer, post-operative infection risk was greater and more inpatient rehabilitation was required than in patients with normal muscle mass. This contrasts with the results of the current study, in which no significant difference was observed in the mean length of hospital stay related to surgery for patients with sarcopenia compared with those without sarcopenia for either the colorectal cancer or NSCLC groups. Indeed, in the NSCLC patients, there was a significantly greater mean total hospital stay (for all patients, including those who did not have surgery) in those who were not sarcopenic. In addition, in the NSCLC patients, greater SM FF was associated with an increase in the length of hospital stay.

The proportion of males and females in the colorectal cancer and NSCLC groups in this study is consistent with the overall incidence in the UK.^27,28^ Whilst there were slightly more males in the colorectal cancer group than the NSCLC group (reflecting the overall UK male:female ratios of these cancers), which could influence body composition measurements because males and females have different proportions of lean and fat tissue, this difference was not significant. In addition, the length of hospital stay regression analyses for each sex demonstrated that the significant relationships observed between body composition and length of hospital stay were not influenced by a single result for either males or females.

Whilst the presence or absence of sarcopenia did not significantly alter the length of hospital stay (either total or related to surgery) in patients with colorectal cancer, an increased SM index was associated with an increased length of hospital stay related to surgery in this group. The lack of a relationship between sarcopenia and the length of surgical hospital stay may have been influenced by the relatively small numbers of patients with sarcopenia (18/45 patients in the colorectal cancer group and 6/15 patients in the NSCLC group) and the low proportion of patients with NSCLC who underwent surgery.

The findings that patients with NSCLC who were not sarcopenic had a greater total length of hospital stay than those who were sarcopenic is perhaps surprising given that sarcopenia is associated with both an increase in post-operative complications (in patients undergoing lung cancer surgery)^29^ and chemotherapy-related toxicity.^30^ The increased total length of hospital stay for patients with NSCLC who were not sarcopenic may instead reflect the fact that their general health and performance status were better than those with sarcopenia, so they were offered more intensive treatments (such as chemotherapy or surgery) that then resulted in longer hospital stays. Whilst differences in performance status did not reach statistical significance, there was a trend for a lower performance status (*i.e.,* better function) in the non-sarcopenic patients. Furthermore, greater SM FF was associated with an increase in the length of both surgical and total hospital stay in the patients with NSCLC. Despite having a normal muscle volume (*i.e.,* not sarcopenic) patients may have had increased fatty infiltration, resulting in a reduced ‘functional’ muscle volume. This has previously been investigated in patients with neuromuscular diseases where fatty infiltration of muscle results in a reduced volume of muscle that contributes to function, termed the ‘contractile cross-sectional area’.^31^

There was no significant association between body composition and either metastatic status at diagnosis or mortality. This is in contrast to previous studies, for example a meta-analysis of 38 studies of 7843 patients with solid tumours and a study of 3241 patients with breast cancer found that low SM index was associated with poorer overall survival.^32,33^ However, these studies had much longer follow-up times compared to our study and the differences may be related to the small number of patients who died during the follow-up period (seven in each group) and the relatively short follow-up time of 12 months. In addition, a relatively small number of patients had metastatic disease at diagnosis in our study. Future studies could address this with longer follow-up periods and larger numbers of patients.

This study has a number of limitations. MRI scans were acquired at a single time point, meaning that longitudinal, within-subject change in body composition could not be assessed. In future studies, it would be pertinent to acquire MRI scans at multiple timepoints to evaluate how the baseline body composition and *change* in body composition over time might predict clinical outcomes. This may enable improved identification of patients who have the potential to benefit from additional nutritional support, particularly prior to treatment. In a study of 111 patients with colorectal cancer undergoing radiotherapy, dietary counselling and protein supplements both increased energy intake and improved quality of life.^34^ Furthermore, nutritional support has been shown to improve outcomes in patients with head and neck cancers^35^ and oesophageal cancer.^36^ In addition, MRI has the potential to be used in interventional studies assessing change in body composition in cancer patients who are receiving different types of nutritional support.

Whilst patients were recruited prospectively, this study involved retrospective analyses of the imaging, demographic and outcome data. Some demographic data were therefore not available in all patients, for example height and weight, and these patients were excluded from the study. Data on performance status were not available for all patients, who were excluded from these subanalyses, which has the potential to influence comparisons of the mean performance status between the two cancer cohorts and the surgical and non-surgical groups. There were also incomplete data on length of hospital stay for some of the patients who were excluded from these subanalyses. Length of hospital stay was divided into two categories: hospital stay related to surgery and total hospital stay. Further breakdown of these categories with a record of the specific reason for each hospital stay would be beneficial to further understand the relationship between body composition and the length of hospital stay. In addition, data relating to suitability for interventions including chemotherapy and surgery were not available. This would be important to assess in future studies alongside data on length of hospital stay and sarcopenia, in order to further understand the relationship between body composition, in particular sarcopenia, and hospital admissions. Sarcopenia was measured using muscle mass and did not include muscle strength as this was not acquired as part of the main Streamline studies. It would be useful to acquire muscle strength information in future studies as this has been recognised as important in defining sarcopenia in combination with muscle mass.

## Conclusion

In this study, body composition, as measured using MRI and a purpose-built in-house segmentation tool, demonstrated differences in the body composition of patients with colorectal cancer and NSCLC. These differences reflect the different phenotypes of these groups, highlighting both the need for tailored approaches to nutritional support and greater understanding of the relationship between different cancers and body composition. Fat deposition in SM was associated with longer hospital stays in patients with NSCLC. This highlights the potential relationship between fat deposition in skeletal muscle and ‘functional’ muscle mass, whereby muscle mass is anatomically normal but the volume contributing to function is reduced.
